# Impaired branched-chain amino acid metabolism may underlie the nonalcoholic fatty liver disease-like pathology of neonatal testosterone-treated female rats

**DOI:** 10.1038/s41598-017-13451-8

**Published:** 2017-10-13

**Authors:** Álvaro Anzai, Rodrigo R. Marcondes, Thiago H. Gonçalves, Kátia C. Carvalho, Manuel J. Simões, Natália Garcia, José M. Soares, Vasantha Padmanabhan, Edmund C. Baracat, Ismael D. C. G. da Silva, Gustavo A. R. Maciel

**Affiliations:** 1 Laboratorio de Ginecologia Estrutural e Molecular (LIM 58), Disciplina de Ginecologia, Faculdade de Medicina FMUSP, Universidade de Sao Paulo, Sao Paulo, SP 01246903 Brazil; 2 Departamento de Morfologia e Genetica, Disciplina de Histologia e Biologia Estrutural, Universidade Federal de Sao Paulo, Sao Paulo, SP 04023900 Brazil; 3 Department of Pediatrics, University of Michigan, Ann Arbor, Michigan 48109 USA; 4 Laboratorio de Ginecologia Molecular e Proteomica, Departamento de Ginecologia, Universidade Federal de Sao Paulo, Sao Paulo, SP 04024002 Brazil

## Abstract

Polycystic ovary syndrome (PCOS) is frequently associated with non-alcoholic fatty liver disease (NAFLD), but the mechanisms involved in the development of NAFLD in PCOS are not well known. We investigated histological changes and metabolomic profile in the liver of rat models of PCOS phenotype induced by testosterone or estradiol. Two-day old female rats received sc injections of 1.25 mg testosterone propionate (Testos; n = 10), 0.5 mg estradiol benzoate (E2; n = 10), or vehicle (control group, CNT; n = 10). Animals were euthanized at 90–94 d of age and the liver was harvested for histological and metabolomic analyses. Findings showed only Testos group exhibited fatty liver morphology and higher levels of ketogenic and branched-chain amino acids (BCAA). Enrichment analysis showed effects of testosterone on BCAA degradation pathway and mitochondrial enzymes related to BCAA metabolism. Testos group also had a decreased liver fatty acid elongase 2 (ELOVL2) activity. E2 group had reduced lipid and acylcarnitine metabolites in the liver. Both groups had increased organic cation transporters (SLC22A4 and SLC16A9) activity. These findings indicate that neonatal testosterone treatment, but not estradiol, produces histological changes in female rat liver that mimic NAFLD with testosterone-treated rats showing impaired BCAA metabolism and dysfunctions in ELOVL2, SLC22A4 and SLC16A9 activity.

## Introduction

Inappropriate exposure to sex steroids during early life has been shown to program reproductive and metabolic abnormalities in several species^1^. It has been proposed that such insults during fetal or neonatal life might be involved in the development of polycystic ovary syndrome (PCOS) phenotype^2^. PCOS is the most common endocrinopathy in women of reproductive age and is characterized by hyperandrogenism and chronic anovulation^3^. In addition, PCOS is commonly associated with insulin resistance, central adiposity, and metabolic syndrome as well as non-alcoholic fat liver disease (NAFLD)^3^. NAFLD is defined as hepatic fat accumulation in the absence of excessive alcohol intake^4^, that is manifested as deposition of lipid droplets in the hepatocytes^5^. NAFLD is the most prevalent liver disease in Western countries^4^.

Although NAFLD in PCOS is believed to be the consequence of insulin resistance, the mechanisms involved in the development of the liver pathology are unknown. The impact of androgen excess, which is receiving considerable attention relative to the development of reproductive and metabolic abnormalities, on liver function remains to be determined. Recent findings that postmenopausal women with high testosterone are at increased risk for fatty liver disease^6^ support a role for androgen. Most studies investigating the role of testosterone on the NAFLD, both in humans and in rodents, have been undertaken in males, thus making it difficult to translate the impact of female hyperandrogenism in the pathophysiology of the fatty liver disease. In this regard, animal models of PCOS phenotype are useful to investigate the mechanisms underlying the liver pathology and may be of translational relevance to women with PCOS patients.

Metabolomic profiling, the systematic identification and quantification of the small molecule metabolic products (the metabolome) of a biological system (cell, tissue, organ, biological fluid, or organism) is a very powerful tool to investigate mediators of liver pathophysiology in hyperandrogenic states, as it enables a functional readout of cellular biochemistry^7^. Using neonatal testosterone and estradiol- treated rat models, the objective of this study was to parse out the effects of androgen and estrogen in the development liver pathology and identify underlying metabolic pathways.

## Materials and Methods

This study was approved by the Institutional Ethics Committee of Faculdade de Medicina da Universidade de São Paulo (CEP-FMUSP) and complies with the Brazilian and International rules for scientific use of animals^8^. The neonatal testosterone and estradiol-treated models used in this study have been well characterized in terms of their reproductive and metabolic phenotypes^9–13^.

### Experimental Design

Thirty two-days-old female Wistar rats (*Rattus norvegicus albinus*) were used in this study. Pups received single subcutaneous administration of the following compounds to induce different PCOS-like phenotypes: 1.25 mg of testosterone propionate (testosterone group, Testos; n = 10)^9^ or 0.5 mg of estradiol benzoate (estradiol group, E2; n = 10)^10^. Both these two models have been well characterized^13^; the Testos group are characterized by hyperandrogenism, polycystic ovaries, and chronic anovulation while the E2 group only manifest polycystic ovaries, and chronic anovulation. Control rats received only olive oil (vehicle) (control group, CNT; n = 10). Pups were maintained with their dams until weaning (21 days). All animals were maintained under artificial illumination on a light-dark cycle of 12:12 hours; with daylight from 7 a.m. to 7 p.m. at a temperature of 22 °C during the whole experiment. Food and water were given *ad libitum*. The animals were weighed weekly.

To evaluate estrous cycle patterns, vaginal smears were taken from 75 days to 90 days of age and the vaginal cytology assessed following Shorr-Harris staining. Estrous cycle phases were defined by prevalence of nucleated epithelial cells, cornified epithelial cells, and/or leucocytes in the vaginal smears. Proestrus: higher number of nucleated epithelial cells. Estrus: higher number of cornified epithelial cells. Metestrus: presence of nucleated epithelial and cornified cells, and leucocytes in similar number. Diestrus: higher number of leucocytes^14,15^. A normal estrous cycle was defined as one exhibiting all phases (proestrus, estrus, metestrus, and diestrus) in a 4–5 days period. Estrous cycle attributes were evaluated as the percentage of the time in each estrous cycle phase. Estrous cycles were not monitored in Testos and E2 group of rats as they did not manifest vaginal opening, a finding consistent with previous reports^9,12^.

At 90–94 days of age, all animals were anesthetized by intraperitoneal administration of ketamine (50 mg/Kg)/xylazine (5 mg/Kg). Following procurement of blood samples between 11:00 a.m. and 13:00 p.m., livers were removed, and animals were euthanized by decapitation. Controls were euthanized in the estrus phase of the cycle.

### Hormonal Measurements

Serum concentrations of LH, FSH, total testosterone and estradiol were measured using ELISA kits (Ucsn Life Sciences Inc., Wuhan, Hubei, China). The limit of detection for LH, FSH, testosterone and estradiol assays was 0.13 ng/mL, 0.92 ng/mL, 0.05 ng/mL, and 4.86 pg/mL, respectively. Intra-assay and inter-assay coefficients of variations for all assays were <10% and <12%, respectively.

### Liver Histomorphometry

Liver fragments were fixed in 10% buffered formaldehyde for 24 hours, paraffin embedded and sectioned at 4 µm thickness. Following staining with Hematoxylin and Eosin, morphometric analyses were conducted with the aid of an optical microscope (Leica, Wetzlar, Hesse, Germany) coupled with a digital camera system (Leica DFC420). To avoid duplicate counting, three sections, at least 100 µm apart from each other were used for the histological analysis, which was performed at 400X magnification. Number of binucleated cells per mm^2^ was counted in 20 randomly selected fields of each section. The same fields were used to evaluate lipid accumulation and scores assigned on the basis of percentage of hepatocytes with cytoplasmic lipid droplets, as follows: <5% of cells, score 0; 6–20%, score 1; 21–30%, score 2; 31–40%, score 3; and >41%, score 4. The morphometric analysis was performed separately by two observers, using the Leica Application Suite 3.0.0 software.

### Targeted (ESI-MS/MS) Quantitative Metabolomics

Five animals per group were randomly selected for targeted metabolite profiling by electrospray ionization (ESI) tandem mass spectrometry (MS/MS). This was carried out on an independent, fee-for-service basis at the quantitative metabolomics platform from Biocrates Life Sciences AG (Innsbruck, Austria). The experimental metabolomics measurement technique is described in detail by patent US 2007/0004044 (accessible online at http://www.freepatentsonline.com/20070004044.html). Briefly, a targeted profiling scheme was used to quantitatively screen for fully annotated metabolites using multiple reaction monitoring, neutral loss and precursor ion scans. Quantification of metabolite concentrations and quality control assessment was performed with the MetIQ software package (BIOCRATES Life Sciences AG, Innsbruck, Austria). An Excel file was then generated, which contained sample ID, metabolite names and metabolites concentrations. This approach allowed detection of 186 different metabolites (p180 kit BIOCRATES Life Sciences AG, Innsbruck, Austria). The metabolite panel included 40 acylcanitines (ACs), 21 amino acids (AAs), 19 biogenic amines (BA), sum of Hexoses (Hex), 76 phosphatidylcholines (PCs), 14 lyso-phosphatidylcholines (LPCs) and 15 sphingomyelins (SMs). Glycerophospholipids were further differentiated with respect to the presence of ester (a) and ether (e) bonds in the glycerol moiety, where two letters (aa = diacyl, ae = acyl-alkyl) denote that two glycerol positions are bound to a fatty acid residue, while a single letter (a = acyl or e = alkyl) indicates the presence of a single fatty acid residue.

In addition to individual metabolite assessment, metabolites related to specific functions were grouped and analyzed together. Groups of AAs were computed by summing the levels of AA belonging to certain families or chemical structures depending on their functions such as total AA, glucogenic AA, ketogenic AA, branched-chain AA (BCAA), aromatic AA (AAA), and glutaminolysis AA. L-Glutamic acid/L-Glutamine ratio was also calculated. Total ACs were also computed. Groups of lipids, important to evaluate lipid metabolism, were also analyzed by summing total LPC, total PC aa, total PC ae, and total SM.

Enzyme activity based on metabolite ratios were calculated for: carbamoyl phosphate synthetase 1 (CPS1) (urea cycle); carnitine palmitoyltransferase 1 (CPT1), and fatty acid elongase 2 (ELOVL2) (fatty acid metabolism); glucokinase regulator (GCKR) (carbohydrate metabolism); solute carrier family 22 member 4 (SLC22 A4), and solute carrier family 16 member 9 (SLC16A9) (acylcarnitines transport); acyl-CoA dehydrogenase C-4 to C-12 straight chain (ACADM), acyl-CoA dehydrogenase C-2 to C-3 short chain (ACADS), and acyl-CoA dehydrogenase long chain (ACADL) (fatty acid metabolism and mitochondrial function). These ratios were computed following procedures described previously^16^. Details of ratios computed and AA groups created are shown in Supplementary Table S1.

### Statistical Analysis

For metabolomic analysis, data was log transformed and Paretto scaling was applied to all quantified metabolites to normalize the concentration distributions using the web-based analytical pipeline MetaboAnalyst 3.0 (www.metaboanalyst.ca)^17^. Receiver Operating Characteristic (ROC) curves were obtained through Support Vector Machine (SVM), Partial Least Squares-Discriminant Analysis (PLS-DA) and Random Forests as well as Logistic Regression Models to calculate Odds Ratios of specific metabolites. ANOVA followed by post hoc Fisher LSD test was performed to compare differences between groups. To further validate the statistical significance, ROC calculations included bootstrap 95% confidence intervals for the desired model specificity as well as other measures including accuracy and false discovery rates (FDR).

For all measures such as hormonal levels and liver morphometry data, normality of distribution was tested by Shapiro-Wilk test. Differences among groups were analyzed by one-way ANOVA followed by Bonferroni post hoc test for data with normal distribution, and by Kruskal-Wallis with Dunn’s multiple comparisons test for skewed data. Repeated measures ANOVA followed by post hoc Bonferroni test to compare treatment groups was performed to evaluate body weight changes over time. Results are described as Means ± SEM. *P* < 0.05 was considered significant.

## Results

### Body Weight Changes

The rate of weight was higher in Testos (10.6%) and E2 (16.2%) groups compared to control group (*P* < 0.0001 for both comparisons) (Supplementary Fig. S1).

### Estrous Cycle and Hormonal Levels

Control rats cycled normally (Supplementary Fig. S2A) and spent 17.50 ± 1.25% of their time at proestrus, 28.75 ± 1.66% at estrus, 23.75 ± 0.83% at metestrus and 30.00 ± 1.25% at diestrus (Supplementary Fig. S2B). Due to absence of vaginal opening, similar measures were not possible in the treated groups. Testos rats displayed higher levels of LH and testosterone compared to controls (*P* < 0.001). There were no significant differences in hormonal levels between E2 group and controls (Supplementary Table S2).

### Liver Pathology

**Figure 1:**
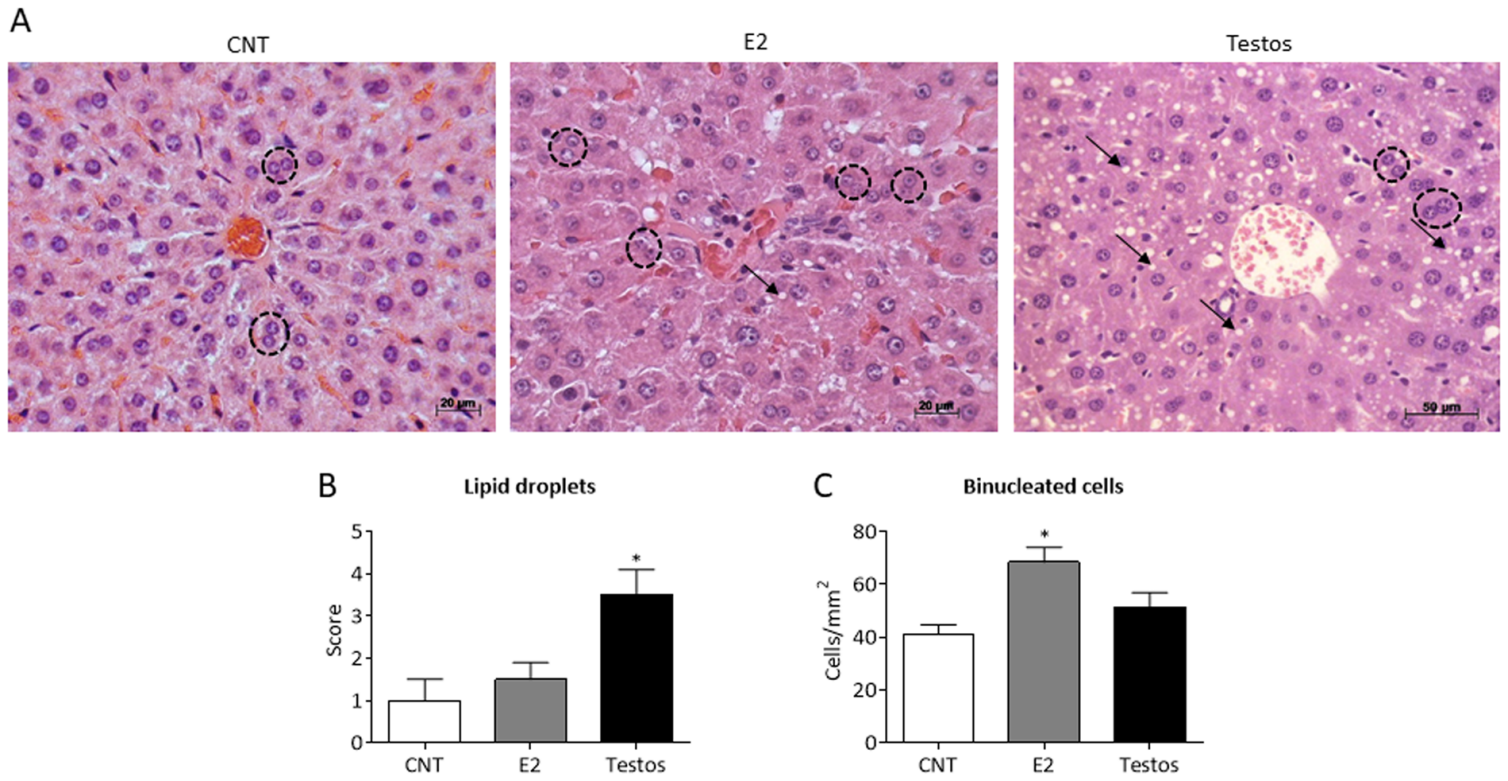
Liver morphology in control (CNT), E2 and Testos groups. Data are shown as mean ± SEM. (**A**) Photomicrographs of liver at 400x magnification (H.E. staining). Black arrows point lipid droplets and black dashed circles indicate binucleated cells. (**B**) Lipid droplets score in hepatocytes. (**C**) Counting of binucleated cells per mm^2^. Asterisks denote significant differences from control.

Histological and morphometric analysis found treatment-specific effects in the liver (Fig. 1A). Testos group manifested greater accumulation of lipid droplets (*P* < 0.02), compared to controls (Fig. 1B). Conversely, the main effect in the E2 group was reflected as increased number of binucleated cells (*P* < 0.03) (Fig. 1C).

### Metabolomic Changes

**Figure 2:**
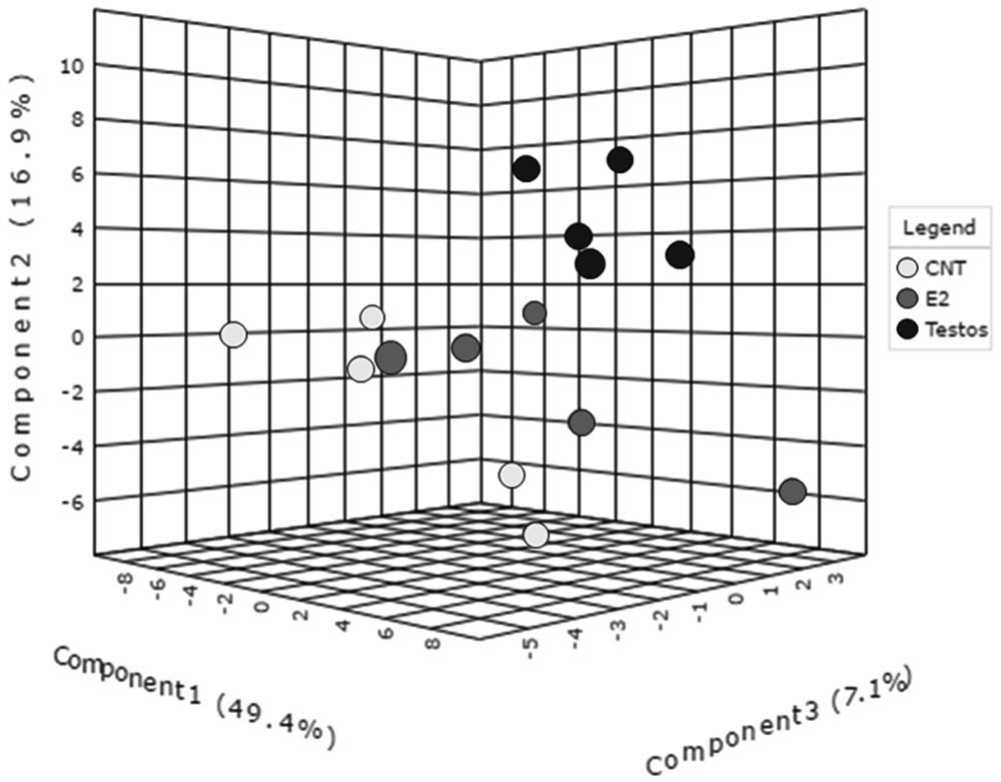
Global metabolome profile of the liver in control (CNT), E2 and Testos groups. 3D score plot among the PLSDA in control, Testos and E2 groups.

**Figure 3:**
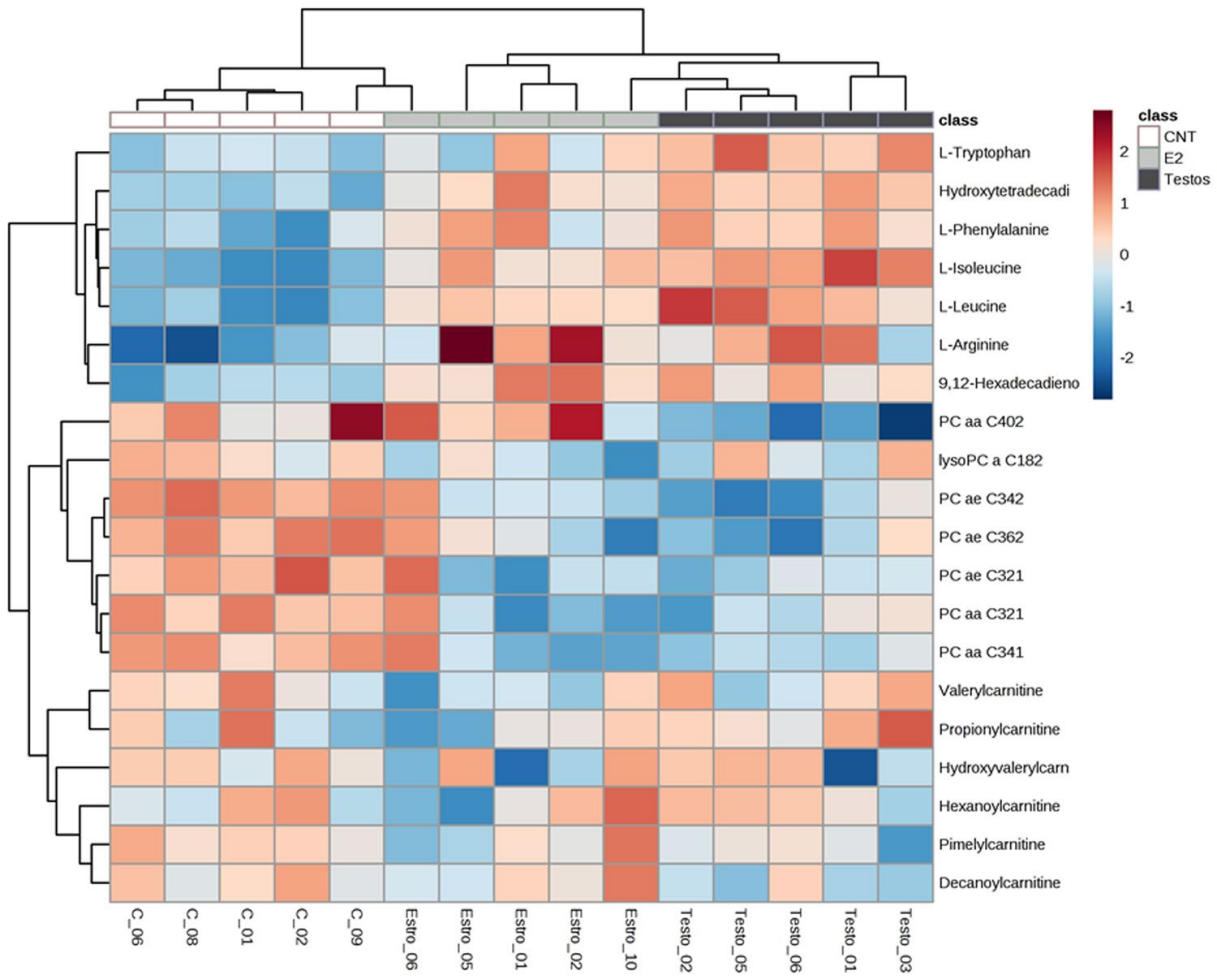
Top 20 metabolites that differ among control (CNT), E2 and Testos groups. Pearson test was performed for cluster analysis.

The first step in metabolome analyses involved global analysis of all the 186 metabolites in the three groups. This approach revealed that the global profiles differed between the 3 groups, with some overlap between E2 and control groups (Fig. 2). Assessment of area under the curve (AUC) of metabolites between control and E2 showed AUC of 0.824 and average of 0.675 (accuracy based on 100 cross validations), indicating these groups do not have a complete segregation of metabolite profiles (Supplementary Fig. S3B). In contrast, AUC analysis revealed a clear segregation in metabolites that differ significantly between control and Testos groups, with AUC of 1 and average accuracy based on 100 cross validations of 1 (Supplementary Fig. S3A). Correlation analysis identified 20 compounds that differentiate controls from E2 or Testos groups, respectively (Fig. 3).

**Figure 4:**
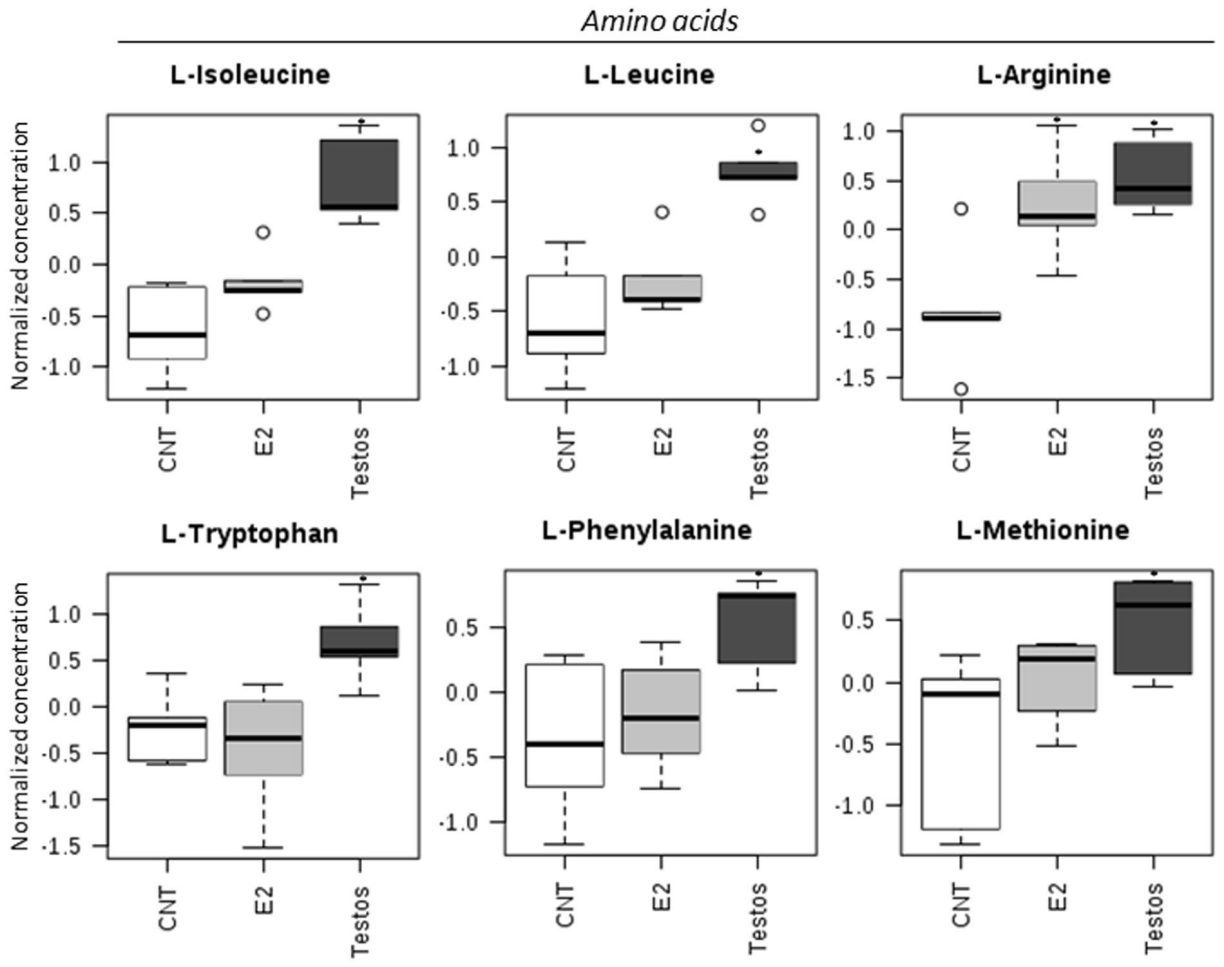
Amino acids that differ significantly between control (CNT), E2 and Testos groups. Asterisks denote significant differences from control.

**Figure 5:**
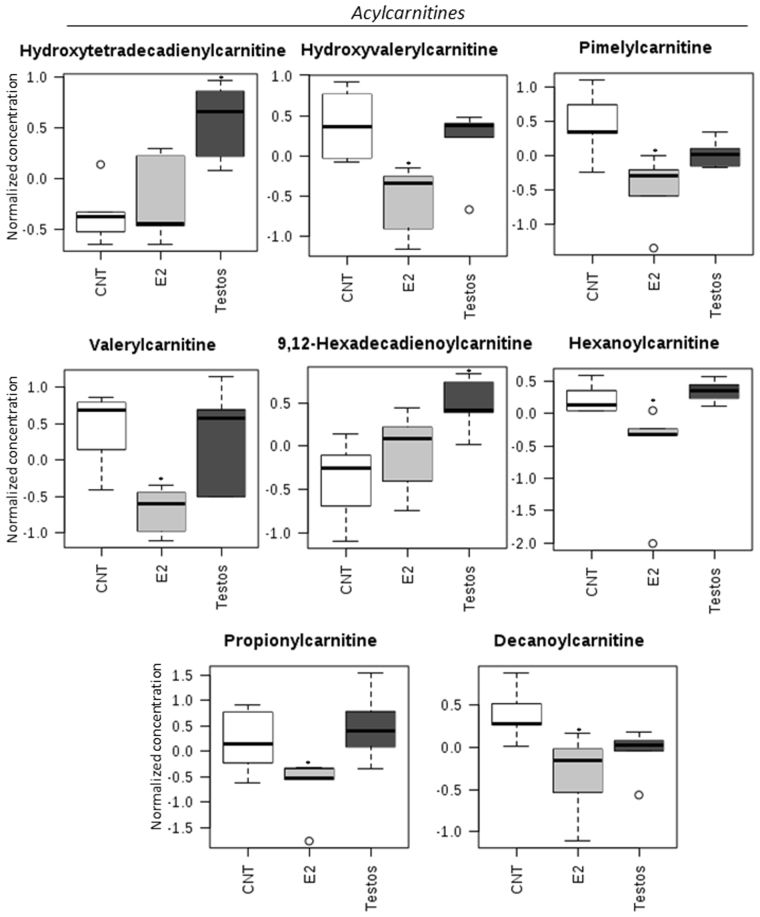
Acylcarnitine metabolites that differ significantly between control (CNT), E2 and Testos. Asterisks denote significant differences from control.

**Figure 6:**
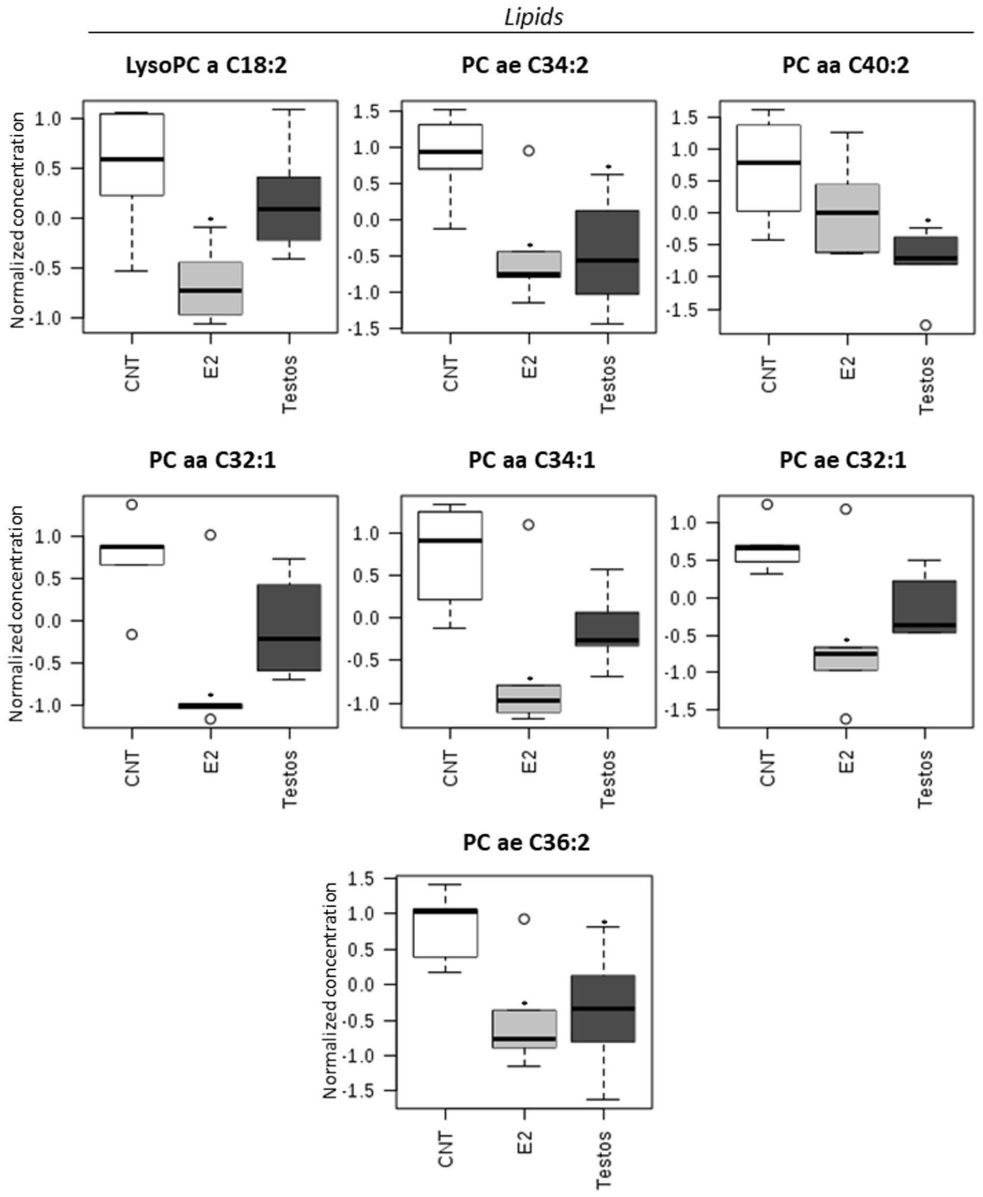
Lipid metabolites that differ significantly between control (CNT), E2 and Testos. Asterisks denote significant differences from control.

The effects of neonatal testosterone and estradiol treatment on the metabolome differed depending on the amino acids, acylcarnitines and lipid measured. Higher levels of certain amino acids (L-isoleucine, L-leucine, L-arginine, L-tryptophan, L-phenylalanine, L-methionine; Fig. 4) and acylcarnitines (hydroxytetradecadienylcarnitine, and 9,12-hexadecadienoylcarnitine; Fig. 5), and lower levels of lipids (PC ae C34:2, PC aa C40:2, and PC ae C36:2; Fig. 6) characterized the Testos group compared with the controls (*P* < 0.05). Barring higher concentrations of L-arginine (Fig. 4), a feature they shared with Testos group, liver from E2 group had lower concentrations of the following acylcarnities (hydroxyvalerylcarnitine, pimelylcarnitine, valerylcarnitine, hexanoylcarnitine, propionylcarnitine, decanoylcarnitine; Fig. 5). At the lipid level, the E2 group also had lower levels of lysoPC a C18:2, PC ae C34:2, PC aa C32:1, PC aa C34:1, PC ae C32:1, and PC ae C36:2 as compared with controls (Fig. 6). Similar direction of changes in E2 and Testos group was evident only at the level of PC ae C34:2 and PC ae C36:2 (Fig. 6).

**Figure 7:**
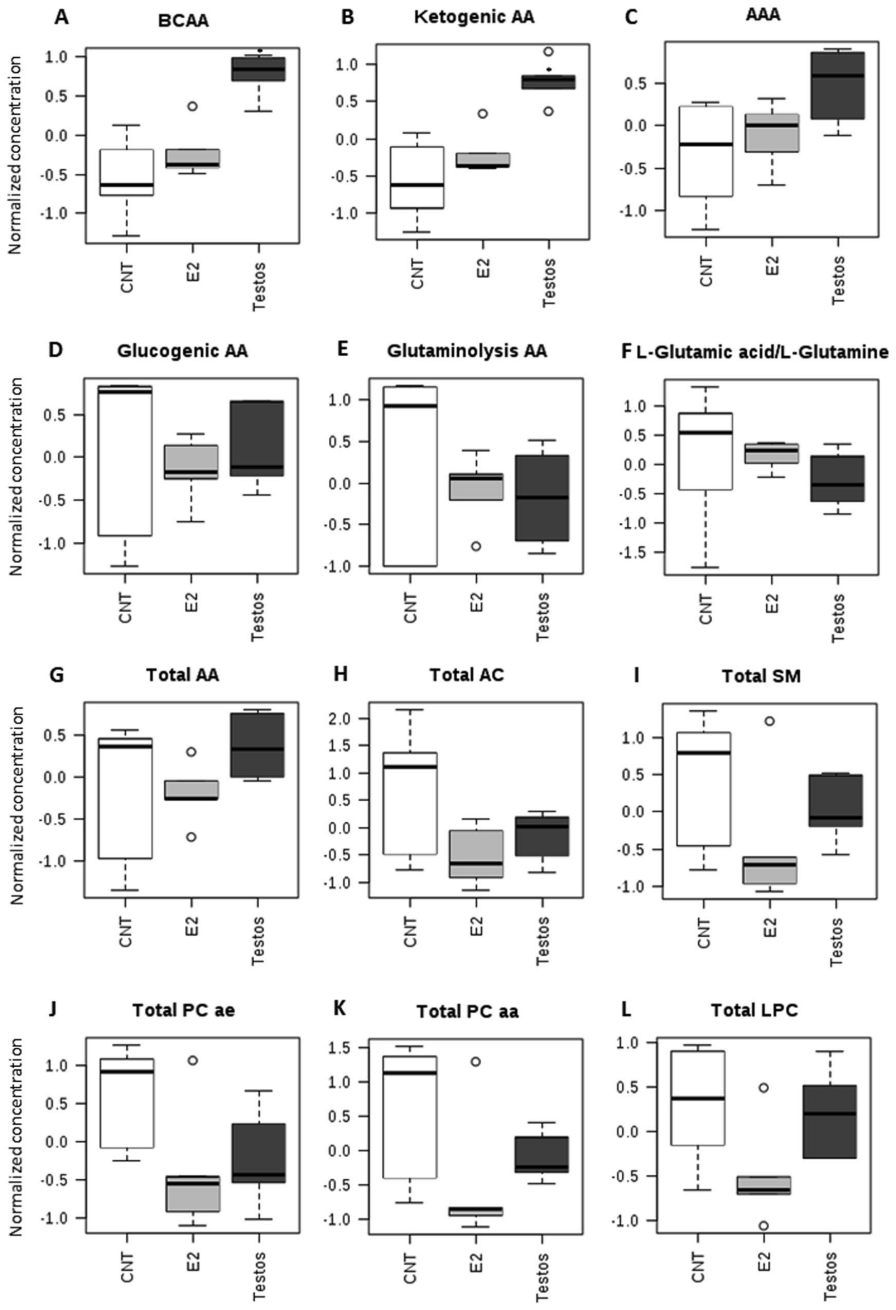
Groups of amino acids (AA) and metabolites that differ significantly between control (CNT), E2 and Testos. (**A**) branched-chain AA (BCAA), (**B**) ketogenic AA, (**C**) aromatic AA (AAA), (**D**) glucogenic AA, (**E**) glutaminolysis AA, (**F**) L-Glutamic acid/L-Glutamine ratio, (**G**) total AA, (**H**) total acylcanitines (AC), (**I**) total sphingomyelins (SM), (**J**) total acyl-alkyl phosphatidylcholines (PC ae), (**K**) total diacyl phosphatidylcholines (PC aa), and (**L**) total lyso-phosphatidylcholines (LPC). Asterisks denote significant differences from control.

When specific functional classes of AA and metabolite groupings (Supplementary Table S1) were compared, there was a significant increase in BCAA (*P* < 0.01) and ketogenic AA (*P* < 0.01) in the liver of Testos group compared with controls (Fig. 7).

**Figure 8:**
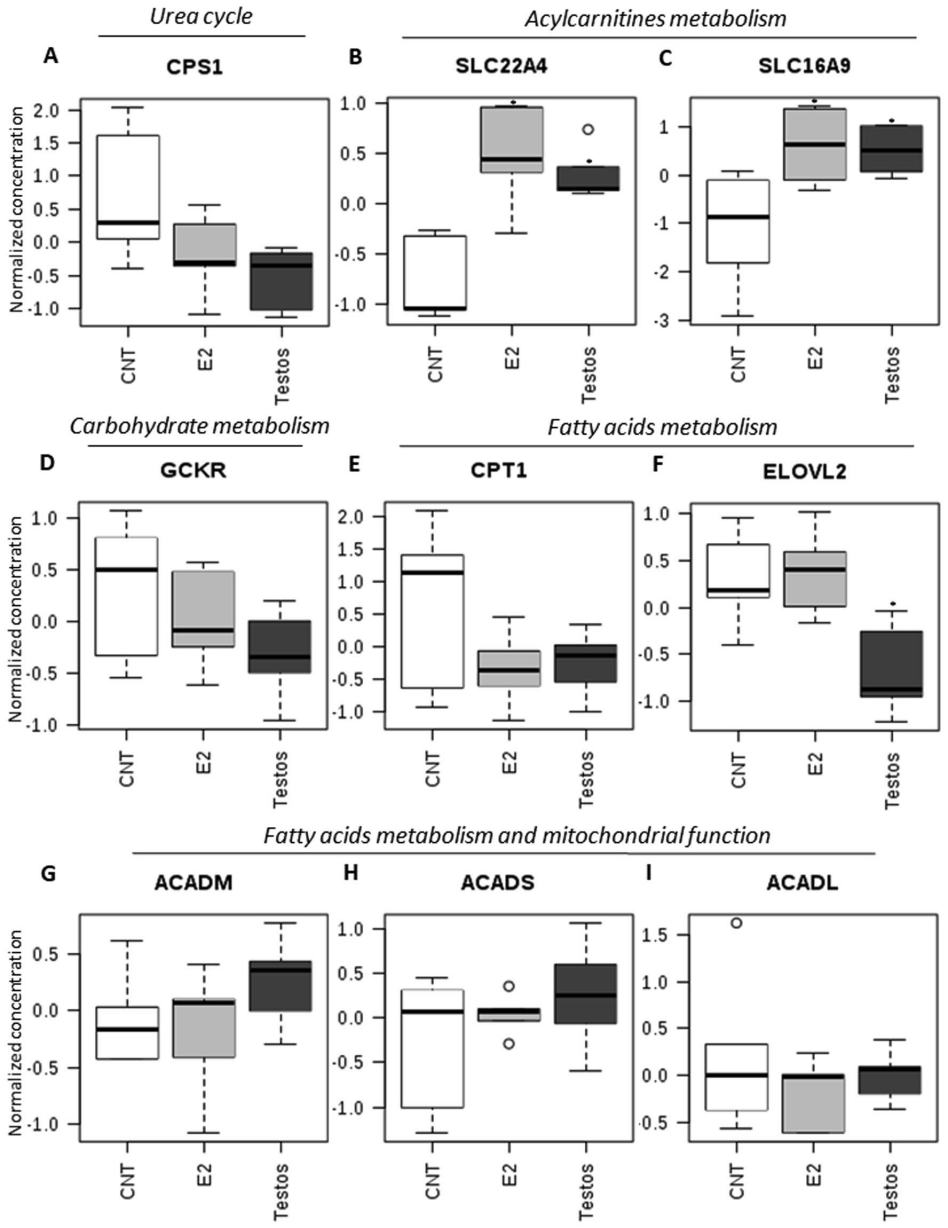
Metabolite ratios related to functions of specific molecules that differ significantly between control (CNT), E2 and Testos. (**A**) carbamoyl phosphate synthetase 1 (CPS1), (**B**) solute carrier family 22 member 4 (SLC22A4), (**C**) solute carrier family 16 member 9 (SLC16A9), (D) glucokinase regulator (GCKR), (**E**) carnitine palmitoyltransferase 1 (CPT1), (**F**) fatty acid elongase 2 (ELOVL2), (**G**) acyl-CoA dehydrogenase C-4 to C-12 straight chain (ACADM), (**H**) acyl-CoA dehydrogenase C-2 to C-3 short chain (ACADS), and (**I**) acyl-CoA dehydrogenase long chain (ACADL). Asterisks denote significant differences from control.

Recently, data from genome-wide association studies coupled with high-throughput metabolic profiling has become powerful tool in the search of insights into genetic variation influences on metabolism in complex disease^18^ in both humans and animal models^16,19,20^. Integration of data from genomic studies, metalobomic profiling and clinical information has provided a system level approach that enables the use of metabolites as proxy of enzymatic activities associated with metabolic traits (Supplementary Table S1). Applying this approach of using metabolite ratios to assess activity of some enzymes and organic cation transporters, we found higher activity of solute carrier family 22 member 4 (SLC22A4) and solute carrier family 16 member 9 (SLC16A9) involved in the transport of acylcarnitines, in the liver of Testos and E2 rats relative to the control group (Fig. 8B,C). In contrast, fatty acid elongase 2 (ELOVL2) activity was lower in Testos group compared with controls (Fig. 8F). There were no significant differences between groups in the activity of carbamoyl phosphate synthetase 1, carnitine palmitoyltransferase 1, glucokinase regulator, acyl-CoA dehydrogenase C-4 to C-12 straight chain, acyl-CoA dehydrogenase C-2 to C-3 short chain, and acyl-CoA dehydrogenase long chain (Fig. 8).

Enrichment analysis performed on Metaboanalyst database to better understand the effects of neonatal testosterone exposure on specific biological processes and enzymes revealed that the pathways for valine, leucine and isoleucine degradation, protein biosynthesis, and betaine metabolism were enriched in Testos rats relative to controls (all *P* < 0.05). In contrast, E2 rats showed enrichments in pathways associated with oxidation of branched chain fatty acids (*P* < 0.05) (Supplementary Table S3). Many mitochondrial enzymes were enriched in Testos group, specifically enzyme related to BCAA metabolism (Supplementary Table S4). In contrast, E2 rats exhibited enrichment in enzymes related to L-arginine metabolism (Supplementary Table S5).

## Discussion

The findings from this study using neonatal testosterone and estradiol-treated rats, two models of PCOS phenotype, revealed Testos group display changes in liver morphometry and metabolome that are strongly associated with NAFLD and impaired BCAA metabolism. The significance of these findings is discussed below.

### Liver pathology

The liver phenotype of the Testos group manifesting higher lipid accumulation mimics the liver phenotype of NAFLD^21^. Studies in animals also indicate that androgens may play a role in the development of fatty liver. For instance sheep exposed to testosterone prenatally presents fatty liver morphology in adult life^22^. Similarly, mice exposed to androgens in prenatal and postnatal life also develop fatty liver morphology^23,24^. A common aspect of all these studies treated with androgens at different time points during their life may relate to their adult hyperandrogenic status. All the models discussed above, including our testosterone-treated model, are functionally hyperandrogenic^1,13,25^. This is supported by the finding that the prevalence of nonalcoholic fatty liver disease in pre-menopausal women is associated with bioavailability of testosterone^26^. Similarly women with hyperandrogenic PCOS are at higher risk to develop nonalcoholic fatty liver disease compared to nonhyperandrogenic PCOS women^27^, and NAFLD prevalence in women with PCOS appear also be related to bioavailability of testosterone^28^. Women with hyperandrogenic PCOS are also at higher risk of metabolic morbidity compared to normoandrogenic phenotype^29^. Taken together, these data suggests hyperandrogenism found in Testos rats might underlie the development of fatty liver morphology. The finding that E2 rats do not present NAFLD phenotype suggests that the development of NAFLD in the Testos rats is a function of androgenic action and not the result of aromatization of testosterone to estradiol.

Occurrence of increased number of binucleated hepatocytes in E2 group may suggest absence of cytokinesis^30^; increased number of binucleated hepatocytes have been shown to occur with the progression of the necro-inflammatory state in the liver^31^. The increase in number of binuclear hepatocytes is not a feature of animal models and humans with NAFLD^32^. This premise is consistent with the histological findings in E2-treated rats, which do not present fatty liver morphology.

### Liver metabolome

The finding that rats treated during neonatal life with testosterone presented higher concentration of BCAA in liver during adult life is consistent with human subjects with NAFLD presenting increased concentration of BCAA in their liver^33^. A number of studies show that circulating levels of BCAA are related to a worsening of the global metabolic profile and to increased insulin resistance^34,35^. Furthermore, circulating BCAA levels are higher in NAFLD subjects with insulin resistance, and an inverse relationship was found to exist between reduced BCAA catabolic activity and circulating BCAA and lipid accumulation in the liver^36^. Enrichment analysis, a tool used to interpret the metabolic impact of early life steroid exposure, also found that the most affected biological processes in the Testos rats was BCAA (valine, leucine and isoleucine) degradation. These findings point to a possible causal relationship among androgen, BCAA metabolism and fatty liver disease. Several studies have pointed to the association of higher BCAA levels and insulin resistance^35,37^. It is hypothesized that the impairment in BCAA metabolism induces mitochondrial dysfunction and insulin resistance-related disorders^35^. Consistent with this premise, Testos rats showed changes in many mitochondrial enzymes. Changes in of BCAA levels, mainly valine and leucine^38^, might also be associated with insulin resistant state of women with PCOS, both lean and overweight/obese^3^. There have been no studies of liver metabolomics in this syndrome to assess if a relationship of BCAA and fatty liver disease exists.

Use metabolites ratios as an index of enzyme activity, revealed differences in activity of acylcarnitine transporters SLC22A4 and SLC16A9 in both the Testos and E2 groups compared to controls. SLC22A4, which is highly expressed in the liver, is a cation transporter involved in the hepatic response to xenobiotic exposure, transportation of antioxidants, elimination of endogenous organic cations, drugs and toxins^39^. SLC16A9 is responsible for carnitine efflux transport and mutations in the gene have resulted in differential immune response in hepatitis B patients^40^. The finding that these organic cation transporters are elevated in both the Testos and E2 rat livers, in the face of Testos liver showing fatty liver pathology while E2 rats not, question a role for these transporters in the development of fatty liver pathophysiology.

In contrast, the decrease in activity of ELOVL2, an enzyme involved in fatty acid metabolism, only in the Testos but not E2 rats suggests the potential for a functional role for this enzyme in the development of NAFLD. The neonatal testosterone-treated rodents, in addition to developing a fatty liver morphology, have been found to display a diabetic-like phenotype^9,10^. Decreased transcriptional expression of ELOVL2 has been described in the liver of diabetic rat models^41^. Interestingly, an increase in methylation, which typically represses the activity of a gene, of liver ELOVL2 in human subjects was found to be associated with aging^42^. Considering that aging in humans is also associated with higher prevalence of type 2 diabetes, nonalcoholic fatty liver disease, and other diseases^42^, it is provocative to speculate a role for the decreased ELOVL2 enzyme activity of Testos rat livers in the development of fatty liver phenotype.

Both Testos- and E2 rats presented higher levels of L-arginine and enrichment of enzymes related to arginine metabolism, suggestive of impaired arginine metabolism. Knockdown of arginase gene leading to impaired arginine metabolism was found to be related to the development of fatty liver in mice^43^. Arginase is an enzyme that converts arginine to ornithine and urea, and the L-arginine to L-ornithine ratio provides an index of arginase activity^44^. However, the trend for an increase in L-arginine/L-ornithine ratio suggestive of reduced arginase activity evident in the Testos rats (Supplementary Fig. S4) may have had no role in the development of NAFLD phenotype, since a similar trend for an increase in L-arginine/L-ornithine ratio was also seen in the E2 group, which did not develop a NAFLD phenotype.

Testos and E2 rats manifest high body weight. However, the contribution of higher body weight to the development of liver pathophysiology and metabolic profile is unclear. While similar changes in metabolite profile in the Testos and E2 rats may be a function of their increased body weight, model-specific changes in Testos and E2 rats may be a function of their different endocrine and metabolic phenotypes. Hyperandrogenism^13^ and increased visceral adiposity^10^ in Testos rats and changes in estrogenic pathways in E2 rats might account for the differences in the liver morphology and metabolites of these two PCOS rat models. To what extent, if any, differences in molar equivalency of testosterone and estradiol dosages used in generating the two models played a role in the differing liver and metabolome phenotype remains to be determined.

To the best of our knowledge, this is the first study characterizing the metabolomic profile in the liver of rat models of PCOS-like phenotype. Because metabolomic profiling can help elucidate mechanisms involved in the pathogenesis and pathophysiology of metabolic diseases^37^ and the limitation in the number of studies profiling liver metabolomics in the NAFLD, the findings from this study are likely to be important in delineating contributors to the development fatty liver disease in a PCOS-like state. Further studies are needed to explore the translational relevance of these findings in humans.

## Electronic supplementary material

Supplementary information
