# Supplementary material for: Impaired branched-chain amino acid metabolism may underlie the nonalcoholic fatty liver disease-like pathology of neonatal testosterone-treated female rats
Source: Sci Rep. 2017 Oct 13;7:13167. doi: 10.1038/s41598-017-13451-8 (PMC5640623; doi:10.1038/s41598-017-13451-8)
Supplement: Supplementary file 1 — Supplementary information [file 41598_2017_13451_MOESM1_ESM.pdf]

Supplementary Information

Impaired branched-chain amino acid metabolism may underlie the nonalcoholic fatty liver disease-like pathology of neonatal testosterone-treated female rats

Álvaro Anzai,[1,+] Rodrigo R. Marcondes,[1,+,*] Thiago H.Gonçalves,[1] Kátia C. Carvalho,[1] Manuel J. Simões,[2] Natália Garcia,[1] José M. Soares Jr,[1] Vasantha Padmanabhan,[3] Edmund C. Baracat,[1] Ismael D. C. G. da Silva,[4] Gustavo A. R. Maciel[1,*]

[1]Laboratorio de Ginecologia Estrutural e Molecular (LIM 58), Disciplina de Ginecologia, Faculdade de Medicina FMUSP, Universidade de Sao Paulo, Sao Paulo, SP 01246903, BR; [2]Departamento de Morfologia e Genetica, Disciplina de Histologia e Biologia Estrutural, Universidade Federal de Sao Paulo, Sao Paulo, SP 04023900, BR; [3]Department of Pediatrics, University of Michigan, Ann Arbor, Michigan 48109, USA; and [4]Laboratorio de Ginecologia Molecular e Proteomica, Departamento de Ginecologia, Universidade Federal de Sao Paulo, Sao Paulo, SP 04024002, BR

[+]These authors contributed equally to this work

***Corresponding authors information:**

Gustavo A. R. Maciel and Rodrigo R. Marcondes. Avenida Dr. Arnaldo, 455, sala 4124, São Paulo-SP 01246903, Brazil. Phone number: +0551130617486. E-mails: gustavo.maciel@hc.fm.usp.br and marcondes_rr@hotmail.com

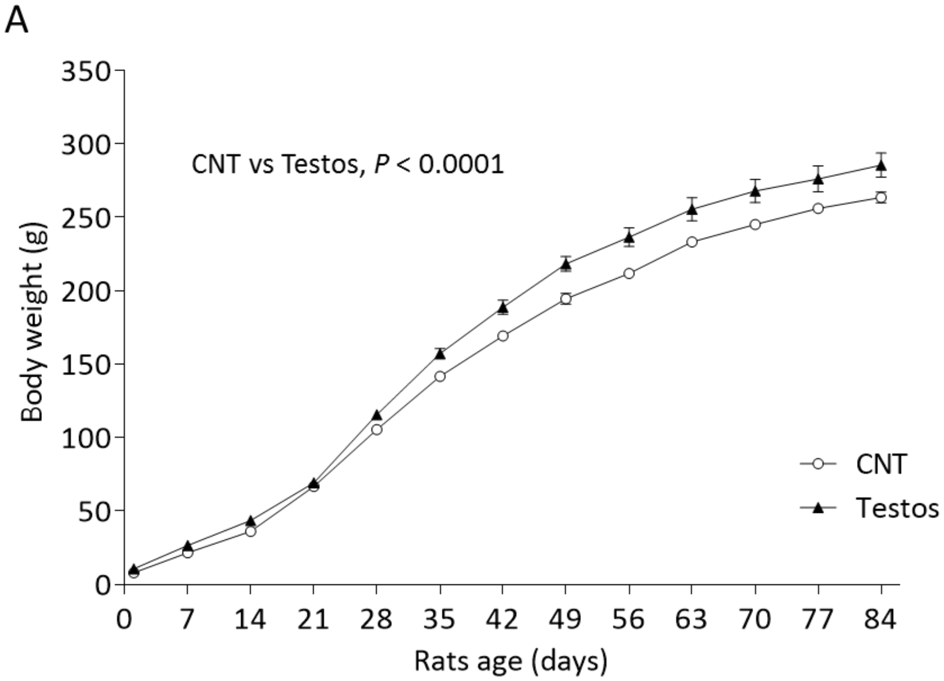

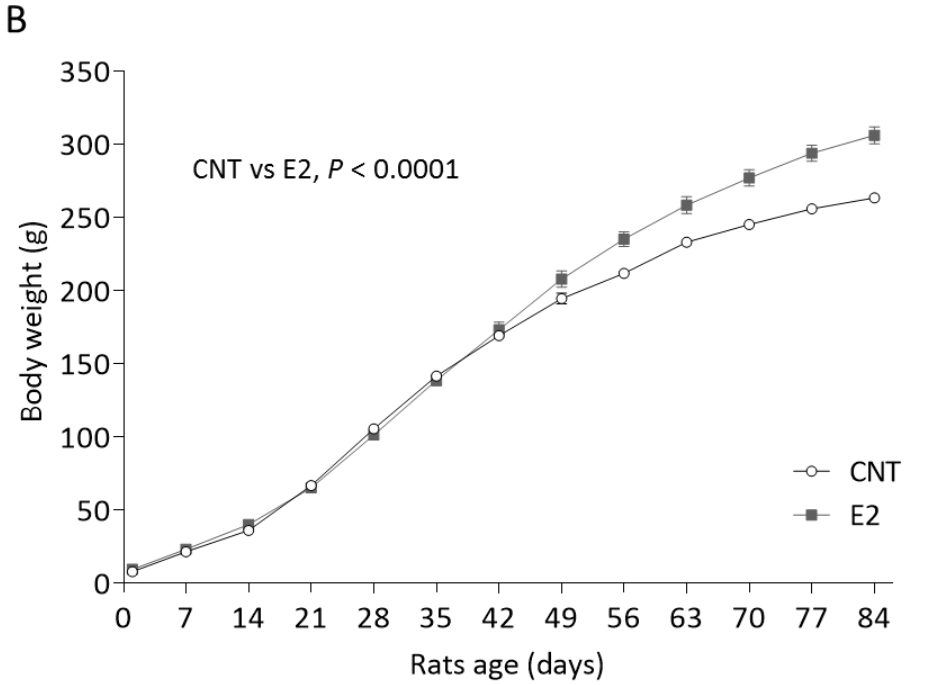

Supplemental Figure S1. Growth curve of animals in control (CNT), E2 and Testos groups. Panel A: CNT vs. Testos, Panel B: CNTvs. E2. Data are shown as mean ± SEM. Asterisks denote significant differences from control.

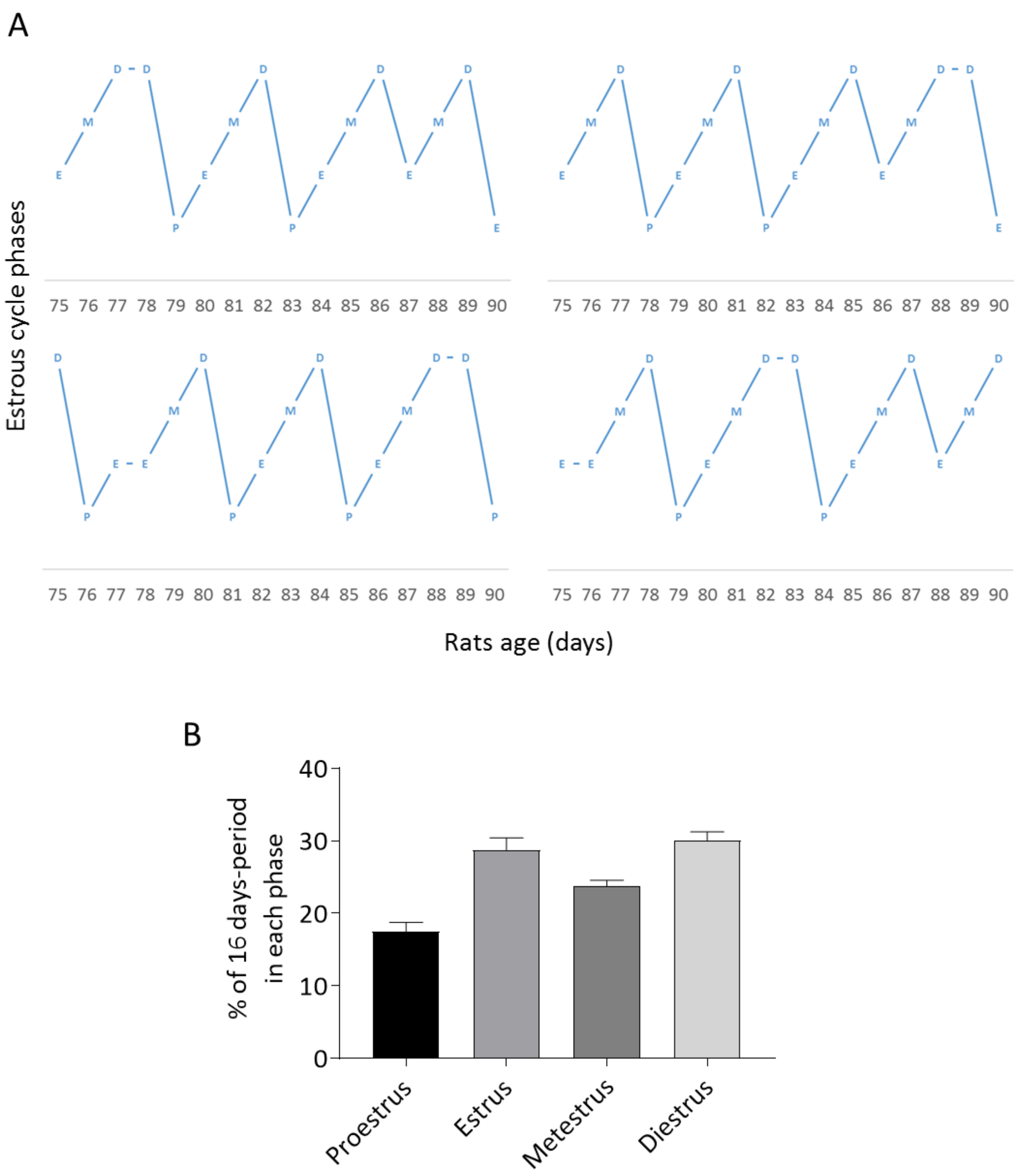

Supplemental Figure S2. Estrous cycle dynamics in control rats. Panel A: Representative patterns of estrous cyclicity in 4 controls. Panel B: Percentage of time spend at each estrous cycle phase. Data are shown as mean ± SEM. P, proestrus; E, estrus; M, metestrus; D, diestrus.

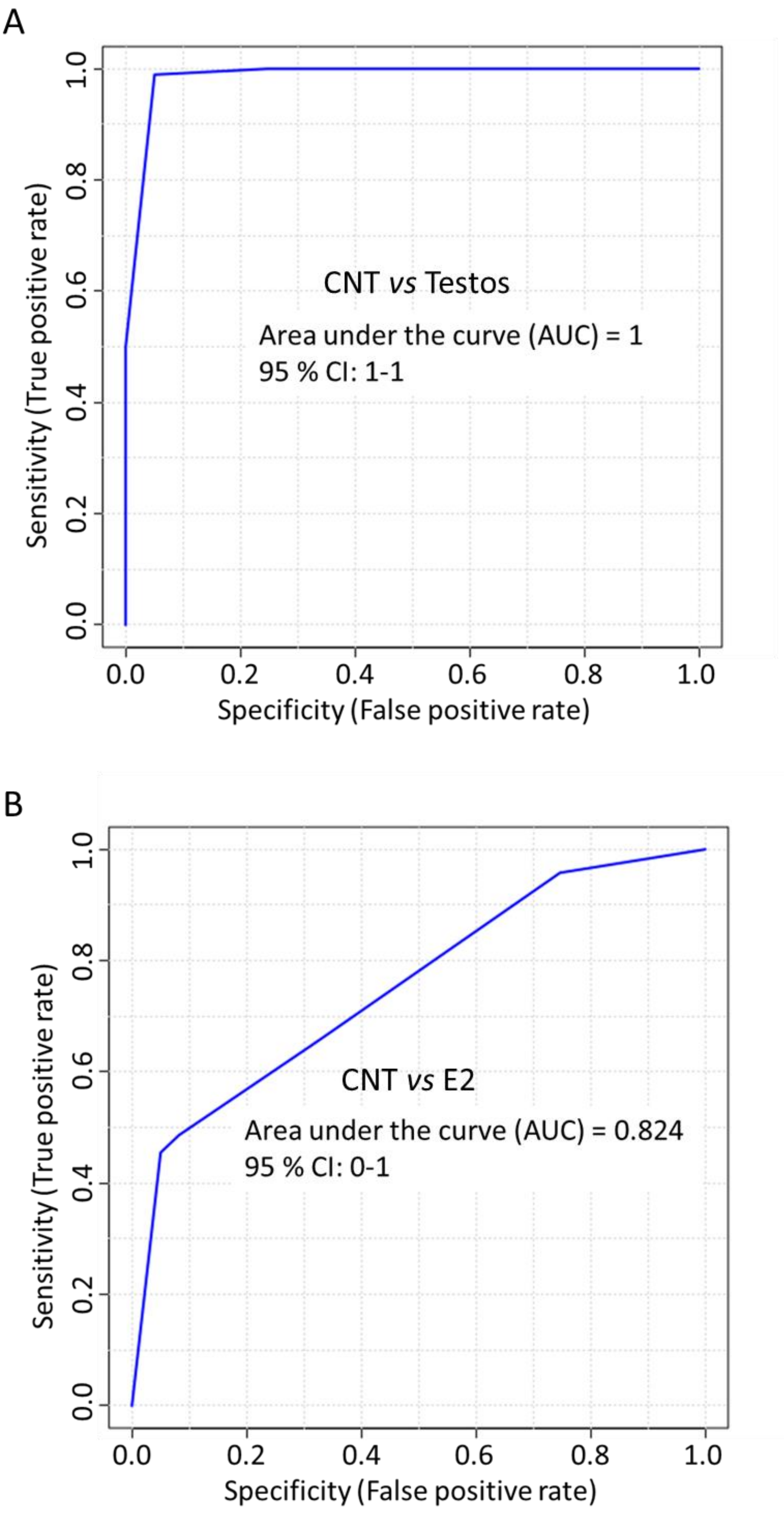

Supplemental Figure S3. Receiver operating characteristic (ROC) curves with area under the curve (AUC) for metabolites the differ significantly between groups. ROC curves with AUC and confidence intervals (CI) values for (A) control (CNT) vs Testos group, and (B) control vs E2 group.

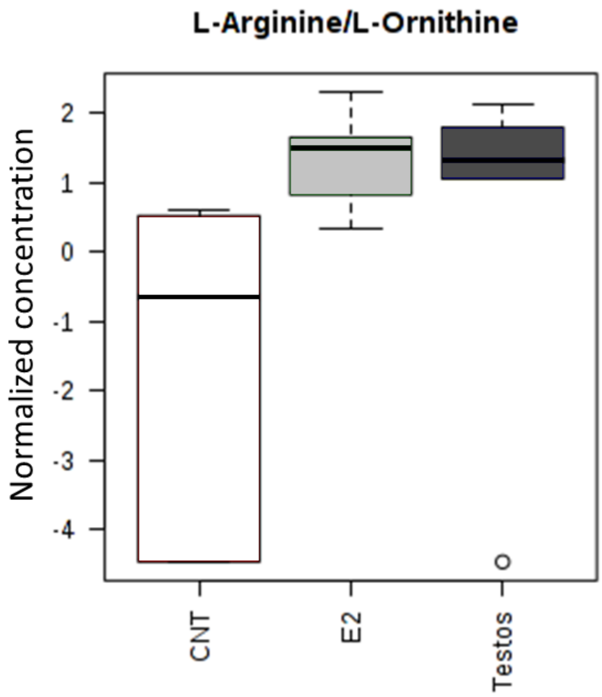

Supplemental Figure S4. L-arginine to L-ornithine ratio (arginase activity).

Supplemental Table S1. Specific amino acid groupings and metabolite ratios for assessment of enzyme activity

**Enzymes and AA groups**	**Formula**
AA groupings	
Aromatic AA (AAA)	Tyr + Phe
Branched-chain AA (BCAA)	Ile + Leu + Val
Glucogenic AA	Ala + Arg + Asn + Asp + Cys + Glu + Gln + Gly + His + Met + Pro + Ser + Val
Glutaminolysis AA	Ala + Asp + Glu
Ketogenic AA	Leu + Lys
Enzyme activity	
Carbamoyl phosphate synthetase 1 (CPS1)	Gly / Arg
Carnitine palmitoyltransferase 1 (CPT1)	C0 / (C16 + C18)
Fatty acid elongase 2 (ELOVL2)	PC aa C40:3 / PC aa C42:5
Glucokinase regulator (GCKR)	PC ae C34:2 / PC aa C32:2
Solute carrier family 16 member 9 (SLC16A9)	Arg / C0
Solute carrier family 22 member 4 (SLC22A4)	Val / C5

Amino acids (AA), Alanine (Ala), Arginine (Arg), Asparagine (Asn), Aspartic (Asp), Cysteine (Cys), Glutamic (Glu), Glutamine (Gln), Glycine (Gly), Histidine (His), Leucine (Leu), Lysine (Lys), Methionine (Met), Proline (Pro), Serine (Ser), Valine (Val), Carnitine (C0), Valerylcarnitine (C5), Hexadecanoylcarnitine (C16), and Octadecanoylcarnitine (C18).

Supplemental Table S2. Gonadotropins and sex steroid levels in control (CNT), E2 and Testos groups

	**CNT (n=10)**	**E2 (n=10)**	**Testos (n=10)**	***P* value**
LH (ng/ml)	11.4 ± 2.3	9.9 ± 1.1	18.4 ± 1.8*	0.001
FSH (ng/ml)	7.5 ± 1.5	6.8 ± 0.8	5.79 ± 0.7	NS
LH/FSH ratio	2.8 ± 1.2	1.6 ± 0.3	3.6 ± 0.5	NS
Testosterone (ng/ml)	3.3 ± 0.4	6.0 ± 1.7	5.4 ± 0.7*	0.01
Estradiol (pg/ml)	5.9 ± 1.6	10.3 ± 1.6	4.9 ± 1.0	NS

Data is presented as mean ± SEM. LH, luteinizing hormone; FSH, follicle-stimulating hormone; NS, not significant. Asterisks denote significant differences from control.

Supplementary Table S3. Enrichment analysis of pathways related to metabome profile in the testosterone and estradiol groups

**Pathway name**	**Total**	**Hits**	***P* value**	**FDR**
Testosterone group				
Valine, leucine and isoleucine degradation	36	3	0.002556	0.089452
Protein biosynthesis	19	17	0.029438	0.43253
Betaine metabolism	10	1	0.037074	0.43253
Estradiol group				
Oxidation of branched chain fatty acids	14	2	0.046058	0.95665

FDR, False discovery rate.

Supplementary Table S4. Enrichment analysis of enzyme activity related to metabome profile in the testosterone group

**Enzyme name**	**Total**	**Hits**	***P* value**	**FDR**
2-Methylprop-2-enoyl-CoA (2-Methylbut-2-enoyl-CoA), mitochondrial	1	1	8,2036E-4	0.014985
2-oxoisovalerate dehydrogenase (acylating; 3-methyl-2-oxopentanoate), mitochondrial	1	1	8,2036E-4	0.014985
3-hydroxyacyl-CoA dehydrogenase (2-Methylacetoacetyl-CoA), mitochondrial	1	1	8,2036E-4	0.014985
acetyl-CoA C-acetyltransferase, mitochondrial	1	1	8,2036E-4	0.014985
acyl-CoA dehydrogenase (2-methylbutanoyl-CoA), mitochondrial	1	1	8,2036E-4	0.014985
Isoleucine mitochondrial transport	1	1	8,2036E-4	0.014985
isoleucine transaminase, mitochondrial	1	1	8,2036E-4	0.014985
L-isoleucine transport via diffusion (extracellular to cytosol)	1	1	8,2036E-4	0.014985
methylmalonyl-CoA mutase	8	1	8,2036E-4	0.014985
Propionyl-CoA carboxylase, mitochondrial	8	1	8,2036E-4	0.014985
2-oxoisovalerate dehydrogenase (acylating; 4-methyl-2-oxopentaoate), mitochondrial	1	1	0.0012048	0.014985
acyl-CoA dehydrogenase (isovaleryl-CoA), mitochondrial	1	1	0.0012048	0.014985
leucine mitochondrial transport	1	1	0.0012048	0.014985

leucine transaminase, mitochondrial	1	1	0.0012048	0.014985
methylcrotonoyl-CoA carboxylase, mitochondrial	1	1	0.0012048	0.014985
methylglutaconyl-CoA hydratase (reversible), mitochondrial	1	1	0.0012048	0.014985
Arginine/Lysine exchanger (Arg in)	1	1	0.0038778	0.040615
L-arginine transport in via sodium symport	1	1	0.0038778	0.040615
L-arginine transport via diffusion (extracellular to cytosol)	1	1	0.0038778	0.040615
5-Hydroxy-L-tryptophan decarboxy-lyase	3	1	0.0087959	0.050011
5-Hydroxy-L-tryptophan exchange	3	1	0.0087959	0.050011
L-Tryptophan,tetrahydrobiopterin:oxygen oxidoreductase (5-hydroxylating)	3	1	0.0087959	0.050011
Serotonin exchange	3	1	0.0087959	0.050011
L-tryptophan transport	1	1	0.0087959	0.050011
3-Hydroxy-L-kynurenine hydrolase	21	1	0.0087959	0.050011
3-hydroxyanthranilate 3,4-dioxygenase	21	1	0.0087959	0.050011
5-hydroxy-L-tryptophan secretion via secretory vesicle (ATP driven)	21	1	0.0087959	0.050011
kynurenine 3-monooxygenase	21	1	0.0087959	0.050011
L-Tryptophan:oxygen 2,3-oxidoreductase (decyclizing)	21	1	0.0087959	0.050011
N-Formyl-L-kynurenine amidohydrolase	21	1	0.0087959	0.050011

nicotinate-nucleotide diphosphorylase (carboxylating)	21	1	0.0087959	0.050011
Quinolinate Synthase (Eukaryotic)	21	1	0.0087959	0.050011
2-aminomuconate reductase	20	1	0.0087959	0.050011
aminomuconate-semialdehyde dehydrogenase	20	1	0.0087959	0.050011
picolinic acid decarboxylase	20	1	0.0087959	0.050011
L-phenylalanine transport via diffusion (extracellular to cytosol)	1	1	0.025719	0.14217
cystathionine beta-synthase	6	1	0.037074	0.17994
cystathionine g-lyase	6	1	0.037074	0.17994
L-methionine transport via diffusion (extracellular to cytosol)	1	1	0.037074	0.17994
Adenosylhomocysteinase	16	1	0.037074	0.17994
methionine adenosyltransferase	16	1	0.037074	0.17994

FDR. False discovery rate.

Supplementary Table S5. Enrichment analysis of enzyme activity related to metabome profile in the estradiol group

**Enzyme name**	**Total**	**Hits**	**P value**	**FDR**
Arginine/Lysine exchanger (Arg in)	1	1	0.024784	0.77695
L-arginine transport in via sodium symport	1	1	0.024784	0.77695
L-arginine transport via diffusion (extracellular to cytosol)	1	1	0.024784	0.77695
fatty-acid--CoA ligase (octanoate)	1	1	0.048465	0.77695
Octanoate transport via diffusion	1	1	0.048465	0.77695

FDR. False discovery rate.
